# The big warp: Registration of disparate retinal imaging modalities and an example overlay of ultrawide-field photos and en-face OCTA images

**DOI:** 10.1371/journal.pone.0284905

**Published:** 2023-04-25

**Authors:** Tobin B. T. Thuma, John A. Bogovic, Kammi B. Gunton, Hiram Jimenez, Bernardo Negreiros, Jose S. Pulido

**Affiliations:** 1 Department of Pediatric Ophthalmology and Strabismus, Wills Eye Hospital, Philadelphia, Pennsylvania, United States of America; 2 Janelia Research Campus, Howard Hughes Medical Institute, Ashburn, Virginia, United States of America; 3 Vickie and Jack Farber Vision Research Center, Wills Eye Hospital, Philadelphia, Pennsylvania, United States of America; 4 Retina Service, Hospital de Olhos do Paraná, Curitiba, Paraná, Brazil; 5 Retina Service, Wills Eye Hospital, Philadelphia, Pennsylvania, United States of America; Yokohama City University, JAPAN

## Abstract

**Purpose:**

To develop an algorithm and scripts to combine disparate multimodal imaging modalities and show its use by overlaying en-face optical coherence tomography angiography (OCTA) images and Optos ultra-widefield (UWF) retinal images using the Fiji (ImageJ) plugin BigWarp.

**Methods:**

Optos UWF images and Heidelberg en-face OCTA images were collected from various patients as part of their routine care. En-face OCTA images were generated and ten (10) images at varying retinal depths were exported. The Fiji plugin BigWarp was used to transform the Optos UWF image onto the en-face OCTA image using matching reference points in the retinal vasculature surrounding the macula. The images were then overlayed and stacked to create a series of ten combined Optos UWF and en-face OCTA images of increasing retinal depths. The first algorithm was modified to include two scripts that automatically aligned all the en-face OCTA images.

**Results:**

The Optos UWF image could easily be transformed to the en-face OCTA images using BigWarp with common vessel branch point landmarks in the vasculature. The resulting warped Optos image was then successfully superimposed onto the ten Optos UWF images. The scripts more easily allowed for automatic overlay of the images.

**Conclusions:**

Optos UWF images can be successfully superimposed onto en-face OCTA images using freely available software that has been applied to ocular use. This synthesis of multimodal imaging may increase their potential diagnostic value. Script A is publicly available at https://doi.org/10.6084/m9.figshare.16879591.v1 and Script B is available at https://doi.org/10.6084/m9.figshare.17330048.

## Introduction

Combining disparate retinal imaging modalities has become increasingly clinically relevant in the diagnosis and management of retinal diseases [1–4]. The combination of functional and structural retinal images has been shown to enhance their interpretation [1]. The Optos camera utilizes a scanning laser ophthalmoscopy (SLO) to capture a 200° ultra-widefield (UWF) image of the retina [5]. This imaging modality provides structural information for a large majority of the retina [5]. Optical coherence tomography angiography (OCTA) has emerged relatively recently as a non-invasive technique for imaging the microvasculature of the retina [6, 7]. OCTA provides both structural and functional data in the management of age-related macular degeneration (AMD), diabetic retinopathy, artery and vein occlusions, glaucoma, and other diseases that affect the retina [6]. Overlaying en-face OCTA images and Optos UWF images could improve the interpretation of both imaging modalities and some companies are now offering this as a separate module.

Image registration, the process of merging two images, is a method for synthesizing retinal information from two different imaging modalities [8]. In image registration, two separate image data sets are transformed into a single coordinate system, which results in the alignment of their positions [8]. However, image registration is difficult [2] especially when two different proprietary systems are attempted to be meshed. Most previous studies on retinal image registration utilize costly software [1, 9–12]. Many ophthalmologists may not be familiar with or have access to this type of software. The algorithms and networks used in such studies may be unavailable or clinically costly and impractical especially in developing countries [1, 9–13]. Thus, we believe it is important to develop a simple, straightforward method of multi-modal proprietary retinal image registration using freely available software.

Feature-based registration uses pairs of common features to register two different images [2]. Previous studies on retinal image registration have often used corresponding landmarks in the retinal vasculature as the basis for feature-based registration [1, 14–16]. Fiji (ImageJ with pre-installed plugins) is a Java-based image processing and analysis program that features plugins for image registration [17]. Fiji is a free software program originally developed by the National Institutes of Health (NIH) and works on MacOS X, Windows, and Linux operating systems [18]. BigWarp is an Fiji plugin for manual pointwise deformable registration [19]. BigWarp has been previously employed to successfully register images of *Drosophila* brain samples and can work where automatic registration does not work and for 3D registration [19]. We therefore wished to see if this powerful image registration tool can be used to register retinal images from different sources.

In this study we demonstrate a simple and effective method to perform true multimodal imaging and show an example of overlaying Optos ultrawide-field (UWF) and en-face OCTA images using the Fiji plugin BigWarp, developed by one of the co-authors (JB). The entire process uses freely available software and does not require familiarity with computer programming or digital image processing. We believe that this method will help ophthalmologists synthesize clinical data from different proprietary sources.

## Materials and methods

This study was approved by the Wills Eye Hospital Institutional Review Board (#2021–87). The need for consent was waived by the ethics committee as the images used in this study were gathered solely for clinical purposes and were immediately anonymized. Diagnostic imaging was performed as part of routine clinical care at Wills Eye Hospital (Philadelphia, PA, USA). Images were collected by a single author (TT) over the course of two weeks from five patients without gross retinal pathology on imaging who had both Optos UWF images (Optos, Inc., Dunfermline, Scotland) and OCTA images (Heidelberg Engineering, Heidelberg, Germany) taken on the same eye on the same day. The OCTA images were uploaded to the Heidelberg Eye Explorer (HEYEX) (Heidelberg Engineering, Heidelberg, Germany) software, which is capable of constructing an en-face OCTA scan. Unfortunately, the HEYEX software was only capable of exporting single images of the OCTA scan at a time. Thus, ten slices of the scan were exported at progressively deeper retinal depths based on the total number of slices in the entire scan. These ten images were saved as PNG files. The Optos UWF image of the same eye was exported as a JPEG file in the OptosAdvance browser-based software program (https://cloud.optos.com/). OptosAdvance was only compatible with the JPEG file format. JPEG is a “lossy” compression file format, which reduces image quality. To minimize this, the images were exported in the highest resolution setting.

Both the Optos UWF image and the first of ten (most anterior) en-face OCTA retinal images were opened in Fiji (ImageJ Version 2.3.0/1.53f) on MacBook Pro or Air laptop computers 2019 or newer running macOS Monterey 12.0.1 (Apple Inc., Cupertino, CA, USA). BigWarp (Version 7.0.4) was then run with both images opened. The Optos UWF image was selected as the moving image and the en-face OCTA image was the fixed image. Ten pairs of reference points were chosen using corresponding vessel branch point landmarks in the retinal vasculature surrounding the macula. The Optos UWF image was then transformed onto the en-face OCTA images using BigWarp’s default thin plate spline method. The resulting transformed (“warped”) Optos UWF image was then exported with the “Moving (warped)” field of view.

A Fiji script (Script A) (accessible at https://doi.org/10.6084/m9.figshare.16879591.v1), written by BigWarp’s author (JB), was developed to align the en-face OCTA images with the resulting transformed Optos UWF image. The script pads all open images so that they align with the field-of-view of the currently selected image, based on its origin. BigWarp stores the origin of the warped image in the image metadata. The resulting padded en-face OCTA images has the same field of view and is aligned with the Optos UWF image.

A second script (Script B) was also written (by TT) to automatically superimpose the en-face OCTA images onto the warped Optos UWF image in Fiji. Script B automatically runs Script A so that all the en-face OCTA photos are correctly padded in the same frame as the Optos UWF image for overlay. It then combines one padded en-face OCTA image with the Optos UWF image into a stack and then converts it into a z-project with maximum intensity. This step is automatically repeated for every en-face OCTA image. Script B then opens each of the ten z-project stacks simultaneously and converted into a single stack. The resulting file is saved as video file in a Tagged Image File Format (TIFF) or Audio Video Interleave (AVI) file format. This process can also be performed manually but is laborious and time-consuming. Script A was folded into Script B, rather than combined into a single script, because they are written in two different programming languages, Groovy and ImageJ Macro language (IJM), respectively.

The process was originally performed by TT. It was then repeated by two other coauthors (JH and BN) after instruction by TT. Each author independently chose corresponding landmarks. Accuracy of transformed and overlayed images were reviewed by TT and JP.

## Results

**Fig 1:**
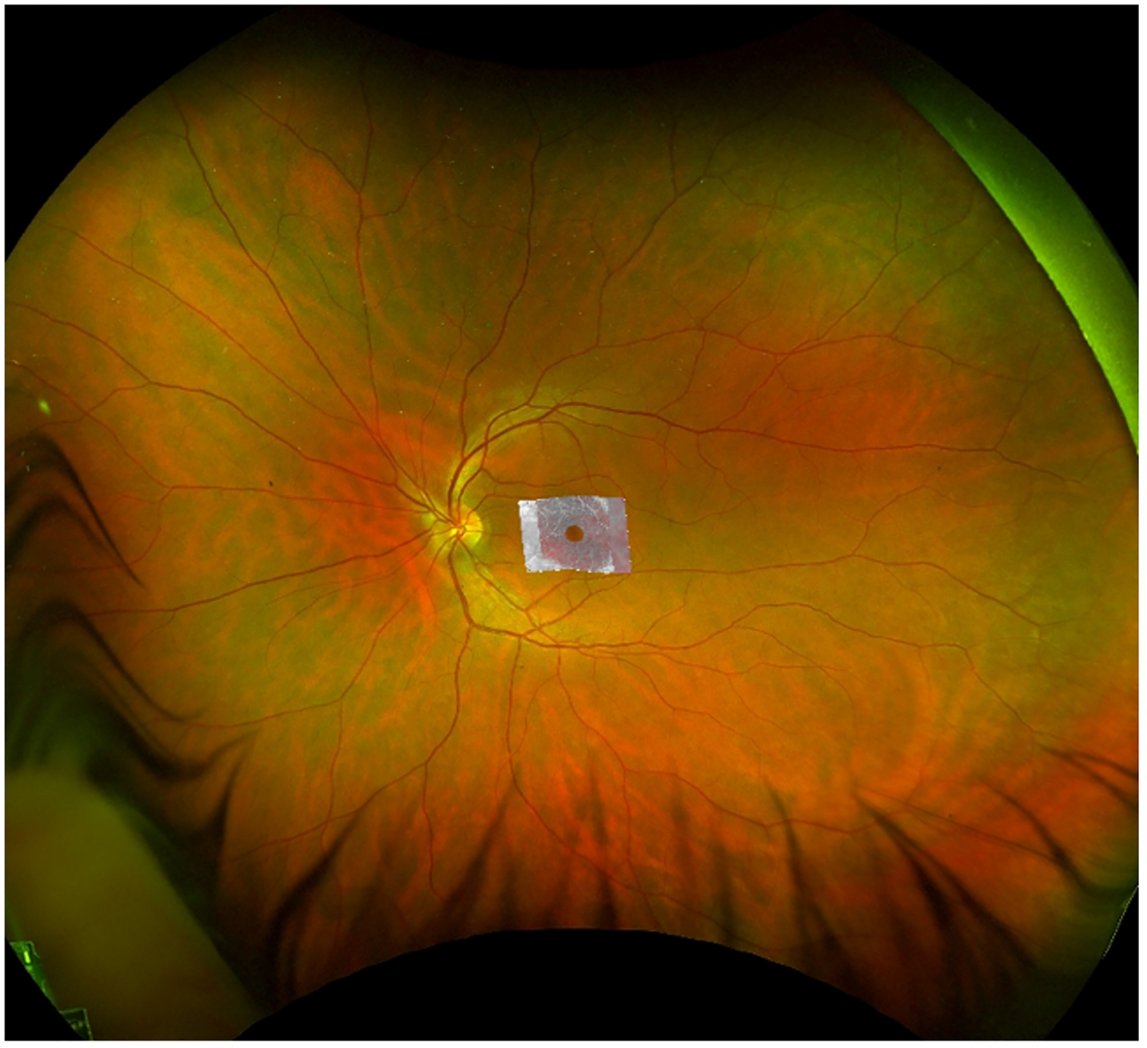
Warping OCTA to UWF. Our first iteration involved warping the en-face OCTA image. This caused undesired distortion and loss of resolution of the macular region image. It was also more time consuming as it required BigWarp be run for every single en-face OCTA image. However, this did not require a padding script as BigWarp automatically padded the smaller en-face OCTA image to properly align with the larger Optos UWF image. This made overlaying the photos much simpler.

BigWarp reliably registered retinal images of different proprietary imaging modalities. In our first iteration, we registered the en-face OCTA image to the Optos UWF image. This method did not require the previously described padding script. The OCTA image, being smaller than the UWF image, was automatically aligned as part of the BigWarp transformation function. However, this was not true of the opposite. Warping a larger image to a smaller image did not automatically align the images for overlay. Regarding overlay success, overlapping blood vessel correspondence demonstrated satisfactory alignment. However, as the OCTA image is much smaller than the UWF image, the OCTA image underwent noticeable resolution loss and distortion (Fig 1). Thus, in the subsequent algorithm we decided to warp the UWF image.

Additionally, the first version of the script required it to be run once for each en-face OCTA image. Thus, an improved script (Script A) was developed to pad all open images to the field-of-view of the selected image. This script only had to be run once for all ten en-face OCTA images, greatly streamlining the process and reducing the time required. Script A was eventually folded into Script B. We developed Script B to automatically perform the function of Script A along with all the Fiji image manipulation work to overlay the images in a stack after running BigWarp. This greatly sped up the process. The run time of script B is around 1–3 minutes and varies depending on the size of the input image files and the computer processing speed. From start to finish the entire process took 6–8 minutes per set of images. Two coauthors, one from Puerto Rico and the other from Brazil, (HJ and BN) were able to independently learn and complete the entire procedure in under 7 minutes.

**Fig 2:**
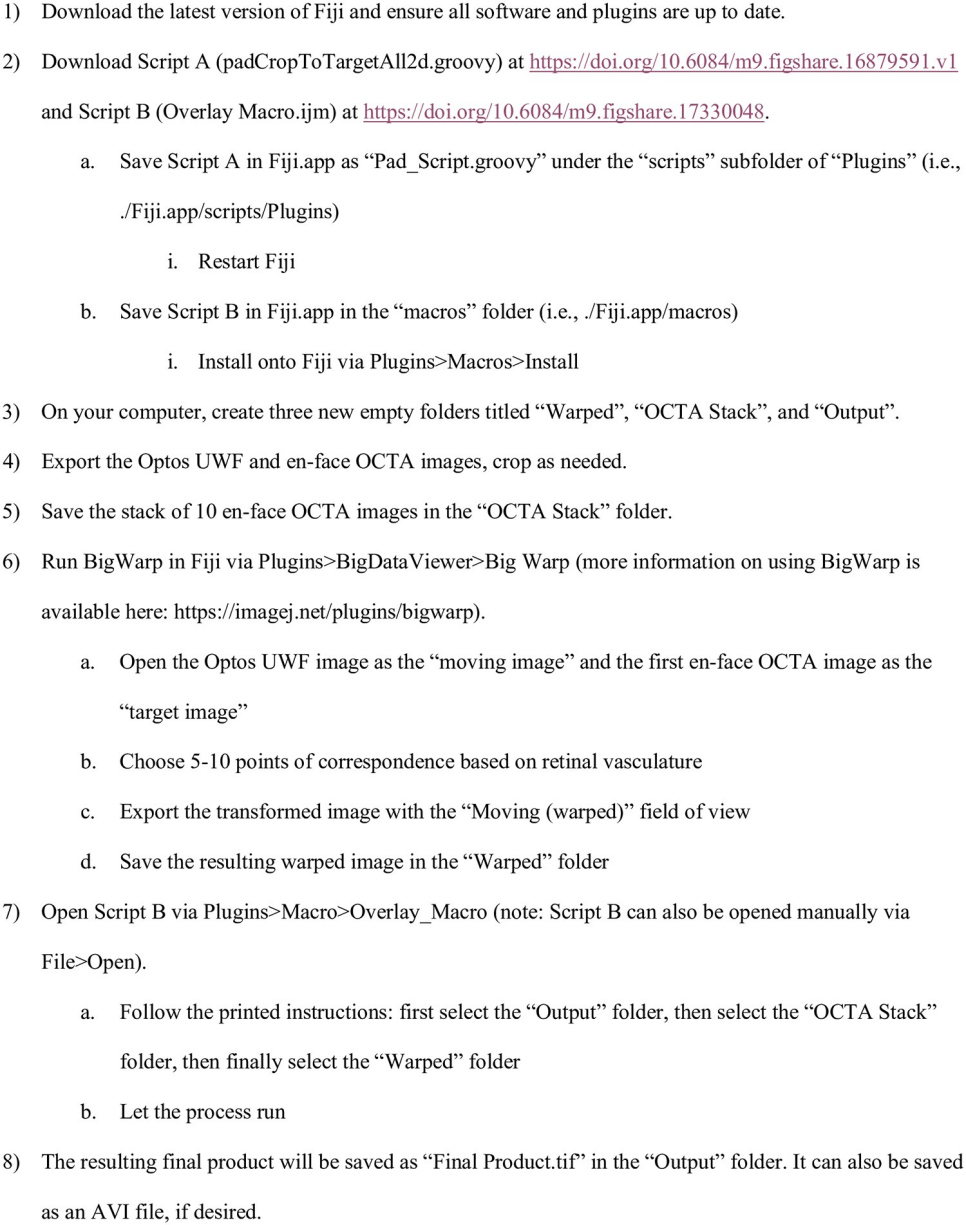
BigWarp overlay algorithm. Method for registering and overlaying en-face OCTA and Optos UWF retinal images using Fiji and BigWarp.

**Fig 3:**
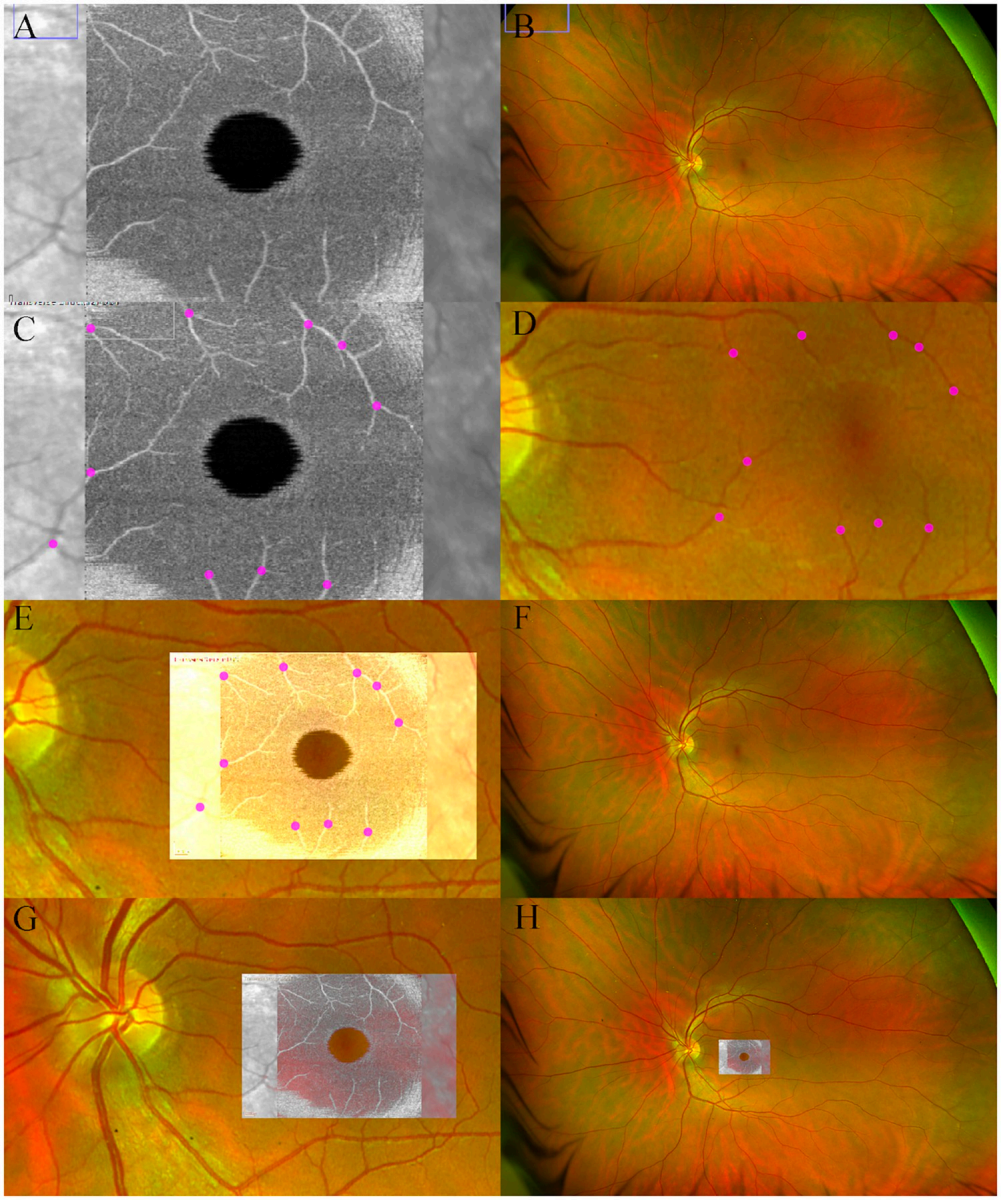
Warping process. Optos UWF image warped onto the en-face OCTA image. We were able to properly pad and align the smaller en-face OCTA image with a user-friendly script designed for this purpose. A) Original en-face OCTA image one of ten in the series. B) Original Optos UWF image. C&D) Pink markers indicate reference points used in BigWarp for image transformation. E) Preview of the en-face OCTA overlayed on the transformed Optos UWF image in BigWarp. Vessel alignment demonstrates a good transformation result. F&G) Resulting overlayed images with the Optos UWF image transformed.

Using the algorithm described in Fig 2, BigWarp reliably registered the Optos UWF image to the en-face OCTA images. Correct image overlay alignment positioning of the en-face OCTA image was consistently achieved. With BigWarp running, the first of the ten en-face OCTA images (Fig 3A) was the “target image” and the Optos UWF image was the “moving image” to undergo transformation (Fig 3B). Ten points of correspondence were selected based on the retinal vasculature (Fig 3C & 3D). A preview of the alignment in BigWarp demonstrates satisfactory overlay based on overlapping corresponding retinal vessels (Fig 3E). The warped Optos UWF appears very similar to the unwarped image because only the macular area was transformed (Fig 3F). Script A was run once for all ten en-face OCTA images to align them with the Optos UWF image (not shown). Overlapping retinal vessels demonstrate good alignment in the final overlayed image (Fig 3G & 3H). A video of the overlayed en-face OCTA images and Optos UWF image displays good alignment at progressive retinal depths. The movie proceeds from superficial to deep retinal en-face OCTA images (S1 Video). An instructional video is included in S2 Video.

An algorithm to perform the entire process manually in Fiji without the use of Script B is provided in S1 Fig.

## Discussion

We have described a simple method for the registration of retinal images from different proprietary modalities using Fiji’s (ImageJ) built-in plugin BigWarp. We applied this to overlay en-face OCTA images with Optos UWF images. Our technique is consistent with previous studies that have registered retinal images based on vascular correspondences [1, 2, 14, 15, 20]. However, to our knowledge, ours is the first to describe a method involving exclusively free software that requires little technical expertise with computers. Importantly, there are no proprietary software packages, algorithms, plugins, or databases required to perform this image registration. Also, no knowledge of computer programming is required. The process is also reasonably quick, requiring under 6–8 minutes per set of images.

In a somewhat similar study, Van Velthoven, et al overlayed conventional angiographic and en-face OCT images using ImagePro with a custom made plugin [1]. Their results combined the angiogram’s physiological information with the en-face OCT’s structural information to enhance the interpretation of the images. However, the software package is proprietary and requires licensing thus, creating a significant barrier to entry. Also, the custom-made plugin is not explicitly stated or made openly available. This creates financial and accessibility barriers to the reproducibility of this technique. Golkar, et al recently described an algorithm to register fluorescein angiography images with en-face OCT images [2]. To do so, they used Matlab, which also requires a software license and writing code in C++, a computer programming language. These aspects create potential obstacles to their accessibility by clinicians. Other proprietary software exists that allows for automatic image registration and mosaicking [21–23]. However, this software does have a fee and it is unclear whether its feature extraction alignment algorithm would function on disparate image modalities that vary greatly in size, resolution, or other such parameters. Of note, Fiji’s built-in automatic feature-based extraction image registration plugin failed when we tried to align the UWF with en-face OCT images. Thus, our method provides a reliable tool for difficult image alignments in addition to being free of charge.

Another advantage of Fiji is that it is actively supported by a large community of experts in imaging and software development [18, 24]. When we ran into a minor issue with BigWarp, we directly contacted the plugin’s developer (JB), who discovered a bug and corrected it promptly. He also developed Script A described herein. Script B was developed by TT based on online guides with minimal previous experience with computer programming. Script B can be edited by users to meet their specific needs. Fiji is a living, constantly evolving platform. Its functionality is always expanding, making it a promising application for developing new techniques in retinal image analysis. Interdisciplinary collaboration with Fiji software developers may help these ideas come to fruition.

There are some limitations to this study. We did not perform formal accuracy assessments of the transformations. However, our goal was not to produce perfect pixel-to-pixel image transformations, but rather to create a set of overlayed images that provide reliable clinical information. In our case, we determined transformation success by comparing the accurate overlap of the course and caliber of the blood vessels in both of the overlayed images. BigWarp can also display an overlay of the warped and target images during the manual landmark selection process, allowing for the user to assess the accuracy of the landmarks and subsequent transformation in real time. If needed, adjustments can then be made. In addition, BigWarp has previously been shown to provide accurate manual registration of microscopic images [19]. Another factor is that there is a learning curve to using Fiji and BigWarp. However, both tools are incredibly useful for scientific image analysis once learned.

We also faced issues with constraints in the software. For one, the HEYEX software was unable to export the entire en-face retinal scan image file and could only export individual en-face images. Thus, we chose to export ten en-face images of the macula at progressively deeper retinal depths. This adds an extra step to the process, increasing time-intensity, and introducing variation in the specific en-face frames selected. Also, the OptosAdvance software could only export images in the JPEG file format, which results in “lossy” compression, reducing image quality [25]. We dealt with this by exporting images on the highest resolution setting. The images had a resolution of 72 dots per inch (DPI). Excessive resolution loss may result in difficulties using the BigWarp procedure as it may obscure identifiable landmarks in the UWF image. Finally, given the small size of the en-face image relative to the UWF image, some of the retinal vessels were difficult to visualize in the UWF image because of the relatively low resolution resulting from intense zoom on the macula. In contrast to BigWarp, there are other proprietary software available that are not free. One is very tied to 2D images, and so cannot represent 3D images or transformations well. This may not matter though if the use case is 2D, of course. Additionally, it is not reproducible. BigWarp makes it easy to save the transformation that is produced and a record of the decisions that were made to produce it (the landmarks). This is especially important when the transformation is non-linear. Some other proprietary software does not make this easy and in some cases not even possible, because that is not its priority—it is design software, not scientific software.

In the future, we would like to improve the speed to complete algorithm, which currently takes around 6–8 minutes per set of images. Script B obviates the need for most of the work to manually perform the image processing after the manual BigWarp transformation. Script B reduces the time required from about 15–20 minutes per set of images to 6–8 minutes. As a result, the main factors that contribute to variance in the time requirements are the file size of the images, the power of the computer, and the familiarity of the user with the process. Nonetheless, two coauthors were able to quickly learn and reproduce the algorithm successfully in under 7 minutes. Moving forward, it would be ideal to develop a software package with a convenient graphical user interface that allows the user to simply drag and drop desired images, manually transform with BigWarp, and automatically overlay them. This will be the goal of future studies.

Herein, we provide a comprehensive, simple guide for overlaying en-face OCTA and Optos UWF images using BigWarp. However, this is just one application of BigWarp. BigWarp stands out as a free tool to successfully perform manual image registration of disparate retinal imaging modalities where automatic registration algorithms may fail. We have seen that certain companies are using proprietary software to similarly do the same. The plugin has broader implications for use as a tool to overlay retinal images in the same plane from nearly any imaging modality, as long as there are clear points of correspondence between the images. For example, BigWarp could potentially be used to overlay any combination of fundus photographs, fluorescein angiography photographs, fundus autofluorescence photographs, en-face OCT images, and en-face OCTA images. The synthesis of these technologies may enhance their interpretation and have important diagnostic value for monitoring patients with complex retinal diseases.

## Supporting information

S1 Fig Algorithm without Script 2. Method for registering and overlaying en-face OCTA and Optos UWF retinal images using Fiji and BigWarp without using Script 2. Script 2 automates steps 5–9 in this algorithm.
(TIF)

S1 Video Final product. (MOV)

S2 Video Instructional video. (MP4)
