# Supplementary figures and images for: The big warp: Registration of disparate retinal imaging modalities and an example overlay of ultrawide-field photos and en-face OCTA images
Source: PLoS One. 2023 Apr 25;18(4):e0284905. doi: 10.1371/journal.pone.0284905 (PMC10129009; doi:10.1371/journal.pone.0284905)

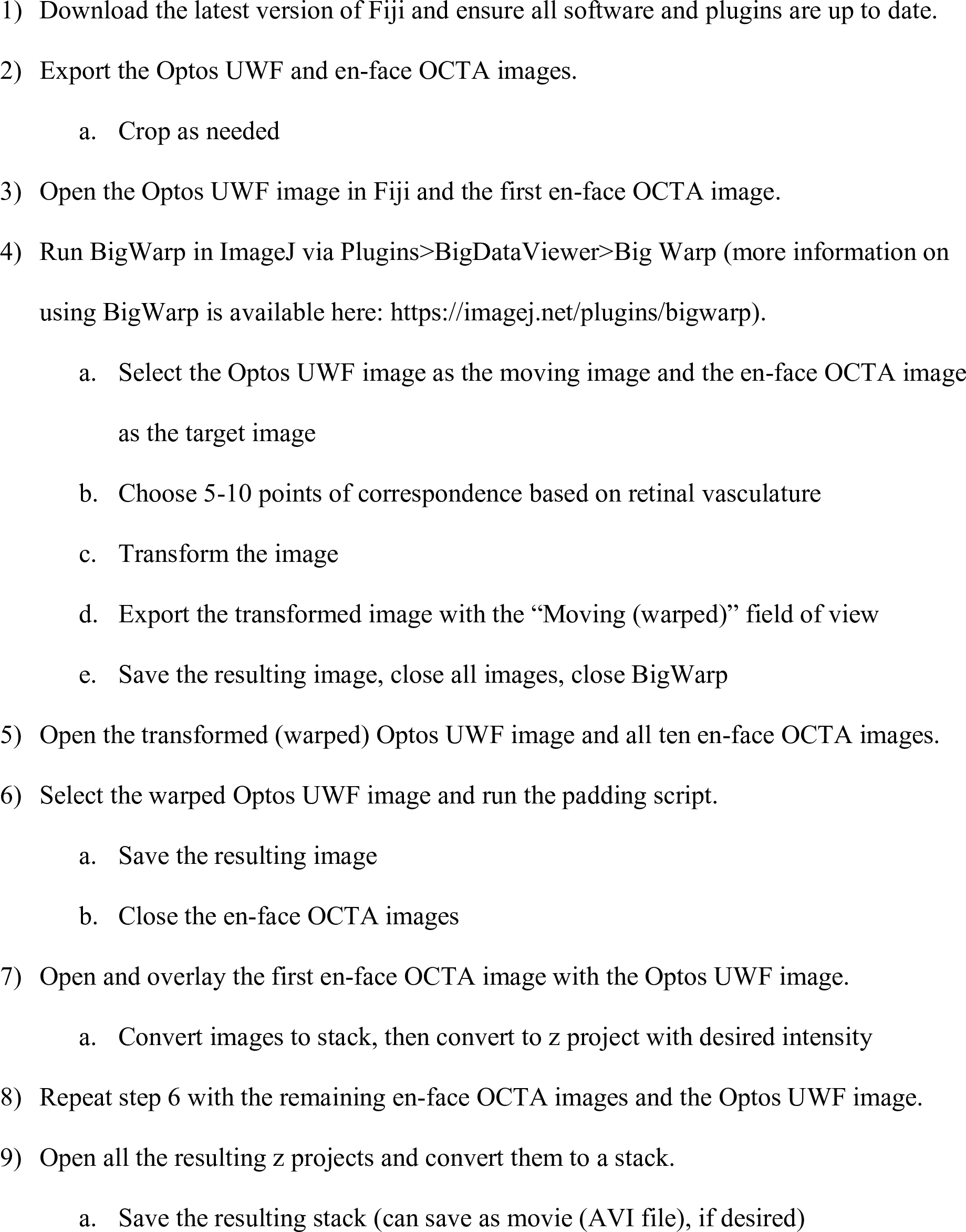

Supplement: S1 Fig — Method for registering and overlaying en-face OCTA and Optos UWF retinal images using Fiji and BigWarp without using Script 2. Script 2 automates steps 5–9 in this algorithm. (TIF) [file pone.0284905.s001.tif]
